# Insulin-like growth factor-1, insulin-like growth factor binding protein-3 and lobule type in the Nurses' Health Study II

**DOI:** 10.1186/bcr3141

**Published:** 2012-03-13

**Authors:** Megan S Rice, Rulla M Tamimi, James L Connolly, Laura C Collins, Dejun Shen, Michael N Pollak, Bernard Rosner, Susan E Hankinson, Shelley S Tworoger

**Affiliations:** 1 Channing Laboratory, Department of Medicine, Brigham and Women's Hospital and Harvard Medical School, 181 Longwood Ave 3rd Floor, Boston, MA, 02115 USA; 2 Department of Epidemiology, Harvard School of Public Health, 677 Huntington Ave Boston, MA, USA 02115; 3 Department of Pathology, Beth Israel Deaconess Medical Center and Harvard Medical School, 330 Brookline Ave Boston, MA, 02115 USA; 4 McGill University, Jewish General Hospital, 3755 Cote St. Catherine Rd, Montreal, Quebec H3T 1E2, Canada

## Abstract

**Introduction:**

Previous research in the Nurses' Health Study (NHS) and the NHSII observed that, among women diagnosed with benign breast disease (BBD), those with predominant type 1/no type 3 lobules (a marker of complete involution) versus other lobule types were at lower risk of subsequent breast cancer. Studies in animal models suggest that insulin-like growth factor-1 (IGF-1) may inhibit involution of lobules in the breast; however, this has not been studied in humans.

**Methods:**

We conducted a cross-sectional study among 472 women in the NHSII who were diagnosed with biopsy-confirmed proliferative BBD between 1991 and 2002 and provided blood samples between 1996 and 1999. A pathologist, blinded to exposure status, classified lobule type in normal adjacent tissue on available biopsy slides according to the number of acini per lobule. For each participant, the pathologist determined the predominant lobule type (that is, type 1, type 2, or type 3) and whether any type 1 or any type 3 lobules were present. Lobule type was then classified as: predominant type 1/no type 3 lobules, which is suggestive of complete involution; or other lobule types. Multivariate logistic models were used to assess the associations between plasma IGF-1, insulin-like growth factor binding protein-3 (IGFBP-3), and the ratio of IGF-1:IGFBP-3 levels with lobule type.

**Results:**

In univariate analyses, greater age, higher body mass index, postmenopausal status, nulliparity, and lower IGF-1 levels were associated with predominant type 1/no type 3 lobules (*P *< 0.05). In multivariate models adjusting for age and assay batch, higher IGF-1 levels were associated with decreased odds of predominant type 1/no type 3 lobules (odds ratio quartile 4 vs. quartile 1 = 0.37, 95% confidence interval = 0.15 to 0.89). Greater ratios of IGF-1:IGFBP-3 levels were also associated with decreased odds of predominant type 1/no type 3 lobules (odds ratio quartile 4 vs. quartile 1 = 0.26, 95% confidence interval = 0.11 to 0.64). These results were slightly attenuated after adjustment for other potential predictors of lobule type.

**Conclusions:**

Higher IGF-1 levels and a greater IGF-1:IGFBP-3 ratio were associated with decreased odds of having predominant type 1 lobules/no type 3 lobules among women with proliferative BBD in the NHSII. This study provides further evidence for the role of insulin-like growth factors in the structure of breast lobules and lobular involution.

## Introduction

Breast tissue in women undergoes transformation throughout puberty, pregnancy, and breastfeeding, and with aging. During puberty, type 1 immature lobules differentiate into type 2 lobules, which further differentiate into type 3 lobules during pregnancy [1]. Type 4 lobules with milk-producing acini are present during lactation, but subsequently undergo involution to type 3 lobules after cessation of breastfeeding [2]. The differentiation of breast lobules during pregnancy and lactation may explain, in part, why these factors are associated with a lower risk of breast cancer [2]. Interestingly, beginning around age 40 an age-related involution also occurs, in which differentiated lobules in the breast regress to earlier structures [1]. Although they appear histologically similar, type 1 lobules in parous women whose breasts have undergone differentiation and involution may be biologically different from those in nulliparous women, and may be less susceptible to carcinogens [2]. In a previous case-control study nested in the Nurses' Health Study (NHS) and the NHSII, having predominant type 1/no type 3 lobules - a marker of complete involution - was associated with a decreased risk of subsequent breast cancer among women with benign breast disease (BBD) [3]. In addition, studies in the Mayo BBD cohort reported that the increasing acini per lobule and no or partial lobular involution were associated with a higher risk of breast cancer [4,5]. However, little is known about potential exposures that may alter the degree of involution/lobule type.

Data from animal studies indicate that insulin-like growth factor 1 (IGF-1) may inhibit involution [6]. IGF-1 is mitogenic and anti-apoptotic, and has been identified as a factor in tumor development and progression [7,8]. Evidence from epidemiologic studies as well as animal studies suggests that elevated IGF-1 levels increase the risk of mammary tumors [9,10]. In addition, IGF-1 levels have been associated with a number of breast cancer risk factors, including birth weight, childhood body size, and mammographic density [11,12]. However, no studies to date have examined the association between IGF-1 levels and involution or lobule type in women. The purpose of this study was to examine the association of IGF-1 and insulin-like growth factor binding protein-3 (IGFBP-3) levels and their ratio with lobule type among women with BBD in the NHSII.

## Materials and methods

### Study population

The NHSII began in 1989, when 116,430 female registered nurses, aged 25 to 42, from 14 US states completed an initial questionnaire. The cohort has been followed by biennially mailed questionnaires to update exposure and covariate information as well as to ascertain incident diseases.

Between 1996 and 1999, 29,611 NHSII members who were free of cancer in 1995 and between 32 and 45 years of age provided a blood sample. Characteristics of this cohort and blood collection details have been previously described [13]. Briefly, premenopausal women who had not taken any type of hormones, been pregnant, or breastfed in the previous 6 months (*n *= 18,521) provided an initial 15-ml blood sample drawn on the third to fifth day of their menstrual cycle (follicular blood draw) and a second 30-ml blood sample drawn 7 to 9 days before the anticipated start of their next cycle (luteal blood draw). The day of the luteal blood collection, the woman shipped both the follicular and luteal blood samples to our laboratory with an ice-pack, via overnight courier, where the samples were processed, separated into plasma, red blood cell, and white blood cell components, and aliquoted into labeled cryotubes.

Women who were ineligible to provide timed samples (that is, perimenopausal, postmenopausal, had undergone a simple hysterectomy, or currently used oral contraceptives or other hormones) or declined to give timed samples (*n *= 11,090) provided a single 30-ml blood sample (referred to as untimed samples). These samples were shipped and processed similarly to the timed samples.

All samples have been stored in the vapor phase of continuously monitored liquid nitrogen freezers (< 130°C) since collection. The study was approved by the Committee on the Use of Human Subjects in Research at the Brigham and Women's Hospital. Informed consent was implied by receipt of completed questionnaires and blood samples.

### Benign breast disease cases

At each questionnaire cycle, participants were asked whether they were diagnosed with BBD and whether the diagnosis was confirmed by biopsy or aspiration. A total of 1,145 women in the blood subcohort reported a first diagnosis of biopsy-confirmed BBD on the 1993 through 2001 questionnaires. Of these 1,145 women, 968 (85% of those eligible) confirmed the diagnosis of BBD and granted permission for review of their biopsy records and pathology specimens. Adequate pathology material was obtained from hospitals and reviewed for 858 women (89% of those who gave permission), and 841 of these (98% of those reviewed) were confirmed by histologic review to be valid BBD cases. Of these, 536 (64%) were classified as proliferative BBD. An additional 64 women were excluded for various reasons, including not having enough plasma or having reported a diagnosis of cancer prior to BBD diagnosis. In total, 472 women with proliferative BBD were included in the current analysis.

### Laboratory assays

IGF-1 and IGFBP-3 levels were assayed in the Department of Medicine and Oncology at McGill University using ELISA with reagents from Diagnostic Systems Laboratory (Webster, TX, USA). IGF-1 was measured in randomly ordered luteal and untimed samples. IGF-1 and IGFBP-3 were assayed for BBD cases diagnosed between 1991 and 1997 as part of a prior study on IGF-1 and proliferative BBD risk [14]. IGF-1 and IGFBP-3 were assayed in a second batch for women diagnosed with proliferative BBD between 1997 and 2002 to incorporate later follow-up cycles and increase the sample size. The mean time between blood collection and biopsy was 3.4 years for the first batch and 1.9 years for the second batch. The medians and ranges of IGF-1, IGFBP-3, and the IGF-1:IGFBP-3 ratio quartiles by batch are presented in Supplemental Table 1 in Additional file 1. The mean coefficients of variation from masked replicate quality control samples included in each batch were 3.5 and 2.8% for IGF-1 and were 1.6 and 3.7% for IGFBP-3.

**Table 1:** Characteristics of study population by lobule type in women with benign breast disease

	No type 1 lobules or mixed lobule types (*n *= 398)	Predominant type 1 and no type 3 lobules (*n *= 74)
Age at biopsy (years)	41.5 (4.6)	44.2 (5.8)
Body mass index (kg/m^2^)	25.6 (5.7)	28.0 (8.0)
Parity (among parous)	2.3 (0.8)	2.4 (1.0)
Age at first birth (among parous)	26.2 (4.1)	26.2 (4.7)
Height	64.8 (2.5)	64.5 (2.2)
Nulliparous	62 (15.6%)	20 (27.0%)
Family history of breast cancer	46 (11.6%)	6 (8.1%)
Ever use oral contraceptive	332 (83.4%)	64 (86.5%)
Menopausal status		
Premenopausal/dubious	343 (86.2%)	54 (73.0%)
Postmenopausal, no PMH	41 (10.3%)	15 (20.3%)
Postmenopausal, PMH	14 (3.5%)	5 (6.8%)
Alcohol		
None	176 (44.2%)	25 (33.8%)
0 to < 1.5 g/day	68 (17.1%)	15 (20.3%)
1.5 to < 4.5 g/day	85 (21.4%)	14 (18.9%)
4.5+ g/day	69 (17.3%)	20 (27.0%)
Age at menarche		
< 11 years	81 (20.4%)	18 (24.3%)
12 years	123 (30.9%)	22 (29.7%)
13 years	113 (28.4%)	19 (25.7%)
14+ years	81 (20.4%)	15 (20.3%)
Histologic categories of benign breast disease		
Proliferative with no atypia	365 (91.7%)	65 (87.8%)
Atypical hyperplasia	33 (8.3%)	9 (12.2%)
Insulin-like growth factor 1		
Quartile 1	93 (23.4%)	24 (32.4%)
Quartile 2	98 (24.6%)	20 (27.0%)
Quartile 3	98 (24.6%)	20 (27.0%)
Quartile 4	109 (27.4%)	10 (13.5%)
Insulin-like growth factor binding protein-3		
Quartile 1	95 (23.9%)	22 (29.7%)
Quartile 2	99 (24.9%)	19 (25.7%)
Quartile 3	101 (25.4%)	17 (23.0%)
Quartile 4	103 (25.9%)	16 (21.6%)

Data from the Nurses' Health Study II (1991 to 2002) presented as mean (standard deviation) or *n *(%). PMH, postmenopausal hormones.

### Lobule type

A study pathologist (JLC), blinded to exposure status, reviewed biopsy slides for the 472 women diagnosed with proliferative BBD who were included in the study. Lobules were classified according to the number of acini per lobule based on Russo and Russo's work [2]. On average, type 1 lobules had fewer than 12 acini, type 2 lobules had approximately 50 acini, and type 3 lobules had approximately 80 acini. The pathologist classified which lobule type was predominant (that is, type 1, type 2, or type 3) and determined whether there were any type 1 or type 3 lobules present for each specimen. For each participant, the lobule type was classified into one of the following two categories: predominant type 1/no type 3 lobules, or other lobule type. These categories were selected based on our previous study that found women with predominant type 1 and no type 3 lobules had a lower risk of subsequent breast cancer compared with women with other lobule types [3]. Biopsy slides were reviewed in two batches: BBD cases diagnosed between 1991 and 1997 (first batch), and BBD cases diagnosed between 1997 and 2001 (second batch). To assess whether lobule-type assessment was consistent across batches, the pathologist reclassified 50 first-batch slides at the time of reading for the second batch. The percentage agreement between the two readings for our two-category lobule-type classification was 78%. ok

### Covariate data

We obtained information on potential correlates of lobule type from biennial NHSII questionnaires. Respondents provided information on age at menarche and height in 1989. Family history of breast cancer was ascertained in 1989 and 1997. Parity, age at first birth, alcohol use, menopausal status, weight, and oral contraceptive use were assessed biennially. For covariates with multiple assessments, we used information from the questionnaire completed closest to BBD biopsy.

### Statistical analysis

We examined the distribution of breast cancer risk factors according to lobule type using univariate logistic regression. For our primary analyses we defined the outcome as predominant type 1/no type 3 lobules. Multivariate logistic regression was used to estimate odds ratios (ORs) and 95% confidence intervals (CIs) for the association between having predominant type 1/no type 3 lobules (vs. other lobule types) and batch-specific quartiles of IGF-1, IGFBP-3, and the ratio of IGF-1:IGFBP-3. Our primary model adjusted for age at biopsy (continuous) and assay batch, with subsequent models additionally adjusting for potential correlates of lobule type including body mass index, menopausal status/postmenopausal hormone use, presence of atypia, parity, alcohol, IGFBP-3 (for the IGF-1 analysis), and IGF-1 (for the IGFBP-3 analysis).

To test for trend, we modeled the square root of IGF-1 levels and the square root of the IGF-1:IGFBP-3 ratio as continuous variables due to their skewed distribution. Untransformed IGFBP-3 levels were modeled linearly to test for trend. We did not detect any statistical outliers for IGFBP-3 levels or the square root of IGF-1 levels or the square root of the IGF-1:IGFBP-3 ratio using the generalized extreme studentized deviate many-outlier approach [15]. In sensitivity analyses, we restricted our study population to parous women and to premenopausal women. Since results did not vary by batch (*P *for interaction > 0.60), all women were combined in the analyses. We used SAS 9.2 software (SAS Institute, Cary, NC, USA) for all analyses and present two-sided *P *values. ok

## Results

Ninety-six (20.3%) women were classified as predominant lobule type 1, 318 (67.4%) were classified as predominant lobule type 2, and 56 (11.9%) were classified as predominant lobule type 3. Study pathologists were unable to classify the predominant lobule type for two women (0.4%). Most women had any type 1 lobules present (*n *= 442, 93.6%) and about one-half had any type 3 lobules present (*n *= 238, 50.4%). Seventy-four of the 472 (16%) women in the study were classified as having predominant type 1/no type 3 lobules (Table 1). These women were older at biopsy and had a higher body mass index than women with other lobule types. In addition, women with predominant type 1/no type 3 lobules were more likely to be nulliparous and postmenopausal.

After adjusting for age and assay batch, women in the highest quartile of IGF-1 had a 63% lower odds of having predominant type 1/no type 3 lobules compared with women in the lowest quartile (OR = 0.37, 95% CI = 0.15 to 0.89; *P*_trend _= 0.01) (Table 2). Additional adjustment for correlates of lobule type and IGFBP-3 slightly attenuated the OR (OR = 0.39, 95% CI = 0.13 to 1.18, *P*_trend _= 0.07). Women in the second and third quartiles of IGF-1 levels had a similar odds of having predominant type 1/no type 3 lobules compared with women in the lowest quartile (OR = 0.92 95% CI = 0.41 to 2.05 and OR = 0.98, 95% CI = 0.41 to 2.31, respectively).

**Table 2:** Predominant type 1/no type 3 lobules according to batch-specific quartiles of plasma IGF-1

	IGF-1 (ng/ml)				
		
	Quartile 1	Quartile 2	Quartile 3	Quartile 4	*P *value
Predominant type 1, no type 3/other lobule type (*n*)	(24/93)	(20/98)	(20/98)	(10/109)	
Age and batch-adjusted^a^	1 (reference)	0.82 (0.40 to 1.67)	0.84 (0.41 to 1.76)	0.37 (0.15 to 0.89)	0.01
Multivariate model 1^b^	1 (reference)	0.98 (0.46 to 2.07)	1.06 (0.49 to 2.29)	0.47 (0.19 to 1.17)	0.08
Multivariate model 2^c^	1 (reference)	0.94 (0.44 to 2.01)	1.00 (0.46 to 2.19)	0.41 (0.16 to 1.04)	0.05
Multivariate model 3^d^	1 (reference)	0.92 (0.41 to 2.05)	0.98 (0.41 to 2.31)	0.39 (0.13 to 1.18)	0.07

Data (vs. other types) from the Nurses' Health Study II presented as odds ratio (95% confidence interval) unless stated otherwise. ^a^Age at biopsy (continuous), insulin-like growth factor 1 (IGF-1) batch. ^b^Age at biopsy (continuous), IGF-1 batch, body mass index (continuous), menopausal status (premenopausal/dubious; postmenopausal, no postmenopausal hormone use; postmenopausal, postmenopausal hormone use), histological category of benign breast disease (proliferative without atypia, proliferative with atypia). ^c^Multivariate model 1 plus parity (nulliparous, parous), alcohol (none, < 1.5, 1.5 to 4.5, 4.5+ g/day). ^d^Multivariate model 2 plus insulin-like growth factor binding protein-3 (continuous).

We observed a stronger association between the IGF-1:IGFBP-3 ratio and lobule type, with women in the highest quartile of IGF-1:IGFBP-3 having a 74% decreased odds of predominant type 1/no type 3 lobules compared with women in the lowest quartile (OR = 0.26, 95% CI = 0.11 to 0.64; *P*_trend _= 0.01) (Table 3). This association was slightly attenuated after adjustment for correlates of lobule type (OR = 0.32, 95% CI = 0.12 to 0.83; *P*_trend _= 0.09). Women in the second and third quartiles of IGF-1:IGFBP-3 levels had a nonstatistically significant decreased odds of predominant type 1/no type 3 lobules compared with women in the lowest quartile (OR = 0.83, 95% CI = 0.39 to 1.76 and OR = 0.75, 95% CI = 0.33 to 1.71, respectively).

**Table 3:** Predominant type 1/no type 3 lobules according to batch-specific quartiles of plasma IGF-1:IGFBP-3 ratios

	IGF-1:IGFBP-3 ratio				
		
	Quartile 1	Quartile 2	Quartile 3	Quartile 4	*P *value
Predominant type 1, no type 3/other lobule type (*n*)	(27/90)	(20/98)	(18/100)	(9/110)	
Age and batch-adjusted^a^	1 (reference)	0.68 (0.33 to 1.39)	0.60 (0.29 to 1.26)	0.26 (0.11 to 0.64)	0.01
Multivariate model 1^b^	1 (reference)	0.80 (0.38 to 1.67)	0.75 (0.34 to 1.66)	0.34 (0.13 to 0.86)	0.10
Multivariate model 2^c^	1 (reference)	0.83 (0.39 to 1.76)	0.75 (0.33 to 1.71)	0.32 (0.12 to 0.83)	0.09

Data from the Nurses' Health Study II presented as odds ratio (95% confidence interval) unless stated otherwise. ^a^Age at biopsy (continuous), insulin-like growth factor 1 (IGF-1):insulin-like growth factor binding protein-3 (IGFBP-3) batch. ^b^Age at biopsy (continuous), IGF-1:IGFBP-3 batch, body mass index (continuous), menopausal status (premenopausal/dubious; postmenopausal, no postmenopausal hormone use; postmenopausal, postmenopausal hormone use), histological category of benign breast disease (proliferative without atypia, proliferative with atypia). ^c^Multivariate model 1 plus parity (nulliparous, parous), alcohol (none, < 1.5, 1.5 to 4.5, 4.5+ g/day).

We did not detect a statistically significant association between IGFBP-3 and lobule type (OR top vs. bottom IGFBP-3 quartile = 0.71, 95% CI = 0.33 to 1.51, *P*_trend _= 0.48) (Table 4).

**Table 4:** Predominant type 1/no type 3 lobules according to batch-specific quartiles of plasma IGFBP-3

	IGFBP-3 (ng/ml)				
		
	Quartile 1	Quartile 2	Quartile 3	Quartile 4	*P *value
Predominant type 1, no type 3/other lobule type (*n*)	(22/95)	(19/99)	(17/101)	(16/103)	
Age and batch-adjusted^a^	1 (reference)	0.83 (0.40 to 1.70)	0.75 (0.36 to 1.57)	0.71 (0.33 to 1.51)	0.48
Multivariate model 1^b^	1 (reference)	0.95 (0.45 to 1.99)	0.83 (0.39 to 1.78)	0.67 (0.31 to 1.45)	0.47
Multivariate model 2^c^	1 (reference)	0.87 (0.41 to 1.87)	0.84 (0.39 to 1.83)	0.61 (0.28 to 1.35)	0.39
Multivariate model 3^d^	1 (reference)	1.06 (0.48 to 2.37)	1.17 (0.48 to 2.81)	0.93 (0.36 to 2.41)	0.63

Data (vs. other types) from the Nurses' Health Study II presented as odds ratio (95% confidence interval) unless stated otherwise. ^a^Age at biopsy (continuous), insulin-like growth factor binding protein-3 (IGFBP-3) batch. ^b^Age at biopsy (continuous), IGFBP-3 batch, body mass index (continuous), menopausal status (premenopausal/dubious; postmenopausal, no postmenopausal hormone use; postmenopausal, postmenopausal hormone use), histological category of benign breast disease (proliferative without atypia, proliferative with atypia). ^c^Multivariate model 1 plus parity (nulliparous, parous), alcohol (none, < 1.5, 1.5 to 4.5, 4.5+ g/day). ^d^Multivariate model 2 plus square-root transformed insulin-like growth factor 1 (continuous).

We observed similar associations when we restricted our study population to premenopausal women (*n *= 397) or parous women (*n *= 390). Owing to the smaller sample size, however, the results were not statistically significant (data not shown).

## Discussion

In this analysis of women with BBD, we observed an inverse association between plasma IGF-1 and the IGF-1:IGFBP-3 ratio with predominant type 1/no type 3 lobules. Interestingly, only IGF-1 levels in the highest quartile were associated with a decreased odds of predominant type 1/no type 3 lobules, suggesting a potential threshold effect. However, we observed more of a linear trend for the IGF-1:IGFBP-3 ratio, which may better represent available IGF-1. The results did not materially change when we restricted our analysis to parous women or premenopausal women (Supplemental Tables 2 to 4 in Additional file 1).

Our findings are consistent with a study reporting that transgenic mice with IGF-1 overexpression in mammary tissue had inhibited postlactational involution [6]. *In vivo *studies in animals suggest that inhibition of postlactational involution increases the risk of subsequent mammary tumors [16]. In addition, recent studies in women have found that lobule type or degree of age-related involution is associated with breast cancer risk. For example, in the NHS and the NHSII, women with predominant type 1/no type 3 lobules had a 29% decreased risk of breast cancer compared with women with other lobule types [3]. Another study did not detect an association between lobule type and breast cancer risk, but the methodology used in the two studies differed [17]. For example, Ramakrishnan and colleagues only assessed the predominant lobule type after the diagnosis of breast cancer in the cases, whereas the NHS/NHSII assessed all lobule types before diagnosis [17].

In the Mayo Clinic BBD cohort, increasing acini per lobule was associated with a stepwise increase in breast cancer risk (*P *= 0.0004). For example, women with more than an average of 40 acini per lobule had 11.9 times the odds of developing breast cancer compared with women with 10 or fewer average acini per lobule [5]. In addition, the acinar count was associated with the degree of involution as assessed by a pathologist. Women with complete involution, on average, had 7.7 acini per lobule versus 32 acini per lobule among women with no involution [5]. Further, in this same cohort, women with no involution had a significantly increased risk of breast cancer than women with complete involution when compared with rates in the Iowa SEER [4].

Interestingly, degree of involution has been associated with mammographic density, which itself is a strong risk factor for breast cancer [18,19]. In the Mayo BBD cohort, women with no or partial involution were more likely to have a high density pattern than women with complete involution (*P*_trend _< 0.01), although both density and involution were independent predictors of breast cancer [20]. Similarly, in premenopausal women, IGF-1 levels have been positively associated with percentage density in some studies [11,21,22], but not all [23]. In general, IGF-1 levels have not been associated with percentage density among postmenopausal women [11,21,23,24]. These data, in conjunction with the results from our study, suggest that high IGF-1 levels may inhibit involution in female breast tissue as well as increase mammographic density. Further research is needed to understand the timing of IGF-1 exposure in relation to morphologic changes in breast tissue as well as the inter-relationship between involution and mammographic density.

Our study has several limitations. First, we only have a single measurement of IGF-1 and IGFBP-3 levels, which may not be representative of typical levels. However, the 3-year intraclass correlation coefficient among premenopausal women in the NHSII was 0.70 for IGF-1 and 0.74 for IGFBP-3, irrespective of menstrual cycle phase. These data suggest that a single measurement of IGF-1 and IGFBP-3 is representative of levels over at least a 3-year period. Lobule type was assessed within 3 years of blood draw for 56% of participants and within 5 years of blood draw for 85%; IGF-1 or IGFBP-3 levels at the time of blood draw are therefore probably representative of those at the time of lobule type assessment. Any misclassification of hormone levels should be nondifferential as the laboratory was blinded to lobule type. Another limitation is that we did not directly assess the degree of involution of lobules in the breast, but rather ascertained lobule type from benign biopsy specimens. However, we have previously reported that women with predominant type 1 and no type 3 lobules, who have probably undergone age-related involution, have a lower risk of breast cancer than women who have other lobule types and thus have probably not undergone age-related involution. Lobule type was assessed on benign breast biopsy specimens, which may not be representative of lobule type throughout the breast given the small size of some of the specimens. Ok However, at least one study has shown that the extent of involution is similar across different quadrants of the breast, indicating that predominant lobule type on a single biopsy specimen is likely to be representative of lobule type throughout the breast [25]. However, there is likely to be some misclassification of lobule type. Any misclassification should be nondifferential since pathologists were blinded to IGF-1 and IGFBP-3 levels, potentially attenuating our results. Finally, our study was cross-sectional so we were unable to prospectively assess whether IGF-1 levels measured prior to biopsy predicted the lobule type or degree of involution.

The strengths of our study include the centralized pathology review of benign biopsy specimens, high-quality IGF-1 and IGFBP-3 assays, and detailed adjustment for correlates of lobule type.

## Conclusion

Higher IGF-1 levels as well as greater IGF-1:IGFBP-3 ratios were associated with a decreased odds of predominant type 1/no type 3 lobules among women with BBD in the NHSII. While this study provides important evidence for the role of insulin-like growth factors in the microscopic anatomy of the breast lobules, further research is needed to elucidate the relationship between hormones, such as IGF-1 and IGFBP-3, and age-related lobular involution in women.

## Abbreviations

BBD: benign breast disease; ELISA: enzyme-linked immunosorbent assay; IGF-1: insulin-like growth factor 1; IGFBP-3: insulin-like growth factor binding protein-3; NHS: Nurses' Health Study.

## Competing interests

The authors declare that they have no competing interests.

## Authors' contributions

MSR participated in the design of the study, conducted the statistical analysis, and drafted the manuscript. RMT, BR, she, and SST participated in the design of the study, advised on the statistical analysis, and drafted the manuscript. JLC, LCC, and DS conducted the lobule type readings. MNP conducted the measurements of IGF-1 and IGFBP-3. All authors read, contributed to, and approved the final manuscript.

## Supplementary Material

Additional file 1 **Supplemental Table 1 showing the ranges and medians of IGF-1, IGFBP-3, and the IGF-1:IGFBP-3 ratio quartiles by batch, NHSII**. Supplemental Table 2 showing the odds ratios (95% confidence intervals) of predominant type 1/no type 3 lobules (vs. other types) according to quartiles of plasma IGF-1 among parous women and premenopausal women only, NHSII. Supplemental Table 3 showing the odds ratios (95% confidence intervals) of predominant type 1/no type 3 lobules according to quartiles of plasma IGF-1:IGFBP-3 ratios among parous women and premenopausal women only, NHSII. Supplemental Table 4 showing the odds ratios (95% confidence intervals) of predominant type 1/no type 3 lobules (vs. other types) according to quartiles of plasma IGFBP-3 among parous women or premenopausal women only, NHSII. Click here for file
