# Supplementary material for: Insulin-like growth factor-1, insulin-like growth factor binding protein-3 and lobule type in the Nurses' Health Study II
Source: Breast Cancer Res. 2012 Mar 13;14(2):R44. doi: 10.1186/bcr3141 (PMC3446378; doi:10.1186/bcr3141)
Supplement: Additional file 1 — Supplemental Table 1 showing the ranges and medians of IGF-1, IGFBP-3, and the IGF-1:IGFBP-3 ratio quartiles by batch, NHSII. Supplemental Table 2 showing the odds ratios (95% confidence intervals) of predominant type 1/no type 3 lobules (vs. other types) according to quartiles of plasma IGF-1 among parous women and premenopausal women only, NHSII. Supplemental Table 3 showing the odds ratios (95% confidence intervals) of predominant type 1/no type 3 lobules according to quartiles of plasma IGF-1:IGFBP-3 ratios among parous women and premenopausal women only, NHSII. Supplemental Table 4 showing the odds ratios (95% confidence intervals) of predominant type 1/no type 3 lobules (vs. other types) according to quartiles of plasma IGFBP-3 among parous women or premenopausal women only, NHSII. [file bcr3141-S1.DOCX]

**Supplemental Table 1:**  Ranges and medians of IGF-1, IGFBP-3, and the IGF-1/IGFBP-3 ratio quartiles by batch, Nurses’ Health Study II

|  | **Quartile 1**  **Median**  **(Range)** | **Quartile 2**  **Median**  **(Range)** | **Quartile 3**  **Median**  **(Range)** | **Quartile 4**  **Median**  **(Range)** |
| --- | --- | --- | --- | --- |
| IGF-1 (ng/mL) |  |  |  |  |
| Batch 1 | 139.7  (61.9-165.3) | 194.7  (167.0-214.4) | 243.1  (214.7-267.3) | 312.8  (267.7-507.1) |
| Batch 2 | 143.0  (68.0-158.6) | 184.9  (161.4-200.6) | 227.4  (201.7-248.9) | 273.7  (251.7-355.4) |
| IGFBP-3  (ng/mL) |  |  |  |  |
| Batch 1 | 4277.3  (3016.2-4624.8) | 4865.5  (4632.3-5173.1) | 5520.0  (5174.2-5765.4) | 6225.5  (5782.4-7356.2) |
| Batch 2 | 4311.0  (3453.8-4765.4) | 5141.7  (4768.9-5489.1) | 5793.5  (5491.1-6142.1) | 6589.4  (6171.0-8072.0) |
| IGF-1/ IGFBP-3  Molar Ratio |  |  |  |  |
| Batch 1 | 0.10  (0.05-0.13) | 0.14  (0.13-0.15) | 0.17  (0.15-0.18) | 0.20  (0.18-0.29) |
| Batch 2 | 0.10  (0.06-0.12) | 0.13  (0.12-0.14) | 0.15  (0.14-0.16) | 0.17  (0.16-0.20) |

**Supplemental Table 2:**  Odds ratios (95% confidence intervals) of predominant type 1/no type 3 lobule type (versus other types) according to quartiles of plasma IGF-1 among parous women and premenopausal women only, Nurses’ Health Study II

|  | **IGF-1 (ng/mL)** | | | |  |
| --- | --- | --- | --- | --- | --- |
|  | **Quartile 1** | **Quartile 2** | **Quartile 3** | **Quartile 4** | **p-value** |
| Parous only |  |  |  |  |  |
| N, predominant type 1, no type 3/ other lobule type | (18/82) | (11/81) | (16/83) | (9/90) |  |
| Age and batch-adjusted^a^ | 1 (Ref) | 0.62 (0.26-1.50) | 1.00 (0.44-2.31) | 0.52  (0.20-1.37) | 0.18 |
| Multivariate model 1^b^ | 1 (Ref) | 0.73 (0.29-1.82) | 1.22 (0.50-2.94) | 0.65  (0.24-1.80) | 0.44 |
| Multivariate model 2^c^ | 1 (Ref) | 0.76 (0.31-1.91) | 1.22 (0.50-2.97) | 0.60  (0.22-1.70) | 0.37 |
| Multivariate model 3^d^ | 1 (Ref) | 0.80 (0.31-2.11) | 1.31 (0.49-3.56) | 0.69  (0.19-2.49) | 0.58 |
| Premenopausal only |  |  |  |  |  |
| N, predominant type 1, no type 3/ other lobule type | (12/66) | (16/88) | (17/86) | (9/103) |  |
| Age and batch-adjusted^a^ | 1 (Ref) | 0.95 (0.40-2.21) | 0.98 (0.42-2.32) | 0.46  (0.17-1.23) | 0.08 |
| Multivariate model 1^b^ | 1 (Ref) | 1.13 (0.47-2.71) | 1.24 (0.51-3.04) | 0.60  (0.21-1.68) | 0.25 |
| Multivariate model 2^c^ | 1 (Ref) | 1.16 (0.48-2.81) | 1.29 (0.52-3.18) | 0.60  (0.21-1.71) | 0.28 |
| Multivariate model 3^d^ | 1 (Ref) | 1.20 (0.47-3.05) | 1.35 (0.50-3.62) | 0.65  (0.19-2.26) | 0.41 |

^a^ Age at biopsy (continuous), IGF-1 batch

^b^ Age at biopsy (continuous), IGF-1 batch, BMI (continuous), menopausal status (premenopausal/dubious; postmenopausal, no pmh use; postmenopausal, pmh use), histological category of BBD (proliferative without atypia, proliferative with atypia)

^c^ Multivariate model 1 plus parity (nulliparous, parous), alcohol (none, <1.5, 1.5-4.5, 4.5+ grams per day)

^d^ Multivariate model 2 plus square-root transformed IGFBP-3 (continuous)

**Supplemental Table 3:**  Odds ratios (95% confidence intervals) of predominant type 1/no type 3 lobule type according to quartiles of plasma IGF-1 to IGFBP-3 ratios among parous women and premenopausal women only, Nurses’ Health Study II

|  | **IGF-1 to IGFBP-3 Ratio** | | | | |
| --- | --- | --- | --- | --- | --- |
|  | **Quartile 1** | **Quartile 2** | **Quartile 3** | **Quartile 4** | **p-value** |
| Parous only |  |  |  |  |  |
| N, predominant type 1, no type 3/ other lobule type | (17/76) | (14/84) | (15/86) | (8/90) |  |
| Age and batch-adjusted^a^ | 1 (Ref) | 0.79  (0.34-1.85) | 0.82 (0.35-1.92) | 0.44  (0.16-1.18) | 0.20 |
| Multivariate model 1^b^ | 1 (Ref) | 0.96 (0.40-2.33) | 1.08 (0.43-2.70) | 0.61  (0.21-1.77) | 0.67 |
| Multivariate model 2^c^ | 1 (Ref) | 0.96 (0.39-2.34) | 1.03 (0.40-2.64) | 0.56 (0.19-1.65) | 0.58 |
| Premenopausal only |  |  |  |  |  |
| N, predominant type 1, no type 3/ other lobule type | (14/64) | (14/87) | (17/87) | (9/105) |  |
| Age and batch-adjusted^a^ | 1 (Ref) | 0.70 (0.30-1.63) | 0.82 (0.36-1.90) | 0.35  (0.14-0.92) | 0.11 |
| Multivariate model 1^b^ | 1 (Ref) | 0.89 (0.37-2.17) | 1.09 (0.44-2.70) | 0.47  (0.17-1.32) | 0.41 |
| Multivariate model 2^c^ | 1 (Ref) | 0.93 (0.38-2.27) | 1.13 (0.45-2.84) | 0.49  (0.17-1.37) | 0.43 |

^a^ Age at biopsy (continuous), IGF-1/IGFBP-3 batch

^b^ Age at biopsy (continuous), IGF-1/IGFBP-3 batch, BMI (continuous), menopausal status (premenopausal/dubious; postmenopausal, no pmh use; postmenopausal, pmh use), histological category of BBD (proliferative without atypia, proliferative with atypia)

^c^ Multivariate model 1 plus parity (nulliparous, parous), alcohol (none, <1.5, 1.5-4.5, 4.5+ grams per day)

**Supplemental Table 4:**  Odds ratios (95% confidence intervals) of predominant type 1/no type 3 lobule type (vs. other types) according to quartiles of plasma IGFBP-3 among parous women or premenopausal women only, Nurses’ Health Study II

|  | **IGFBP-3 (ng/mL)** | | | | |
| --- | --- | --- | --- | --- | --- |
|  | **Quartile 1** | **Quartile 2** | **Quartile 3** | **Quartile 4** | **p-value** |
| Parous only |  |  |  |  |  |
| N, predominant type 1, no type 3/ other lobule type | (17/82) | (12/82) | (14/86) | (11/86) |  |
| Age and batch-adjusted^a^ | 1 (Ref) | 0.72 (0.31-1.70) | 0.84  (0.37-1.92) | 0.64 (0.27-1.56) | 0.59 |
| Multivariate model 1^b^ | 1 (Ref) | 0.80 (0.34-1.92) | 0.87  (0.37-2.04) | 0.55 (0.22-1.37) | 0.46 |
| Multivariate model 2^c^ | 1 (Ref) | 0.81 (0.33-1.94) | 0.88  (0.37-2.08) | 0.53 (0.21-1.33) | 0.47 |
| Multivariate model 3^d^ | 1 (Ref) | 0.83 (0.33-2.12) | 0.93  (0.35-2.50) | 0.58 (0.19-1.79) | 0.84 |
| Premenopausal only |  |  |  |  |  |
| N, predominant type 1, no type 3/ other lobule type | (16/80) | (13/82) | (14/89) | (11/92) |  |
| Age and batch-adjusted^a^ | 1 (Ref) | 0.73 (0.32-1.68) | 0.72  (0.32-1.64) | 0.58 (0.24-1.39) | 0.46 |
| Multivariate model 1^b^ | 1 (Ref) | 0.78 (0.34-1.79) | 0.72  (0.32-1.66) | 0.53 (0.22-1.29) | 0.41 |
| Multivariate model 2^c^ | 1 (Ref) | 0.70 (0.30-1.66) | 0.79  (0.34-1.84) | 0.53 (0.22-1.29) | 0.39 |
| Multivariate model 3^d^ | 1 (Ref) | 0.75 (0.30-1.85) | 0.87  (0.33-2.28) | 0.60 (0.21-1.75) | 0.96 |

^a^ Age at biopsy (continuous), IGFBP-3 batch

^b^ Age at biopsy (continuous), IGFBP-3 batch, BMI (continuous), menopausal status (premenopausal/dubious; postmenopausal, no pmh use; postmenopausal, pmh use), histological category of BBD (proliferative without atypia, proliferative with atypia)

^c^ Multivariate model 1 plus parity (nulliparous, parous), alcohol (none, <1.5, 1.5-4.5, 4.5+ grams per day)

^d^ Multivariate model 2 plus IGF-1 (quartiles)
